# Unraveling the genetic spectrum of inherited deaf-blindness in Portugal

**DOI:** 10.1186/s13023-025-03542-5

**Published:** 2025-01-14

**Authors:** Telma Machado, Telmo Cortinhal, Ana Luísa Carvalho, Francisco Teixeira-Marques, Rufino Silva, Joaquim Murta, João Pedro Marques

**Affiliations:** 1 Ophthalmology Department, Centro Hospitalar e Universitário de Coimbra (CHUC), Hospitais da Universidade de Coimbra (HUC), ULS Coimbra, Praceta Prof. Mota Pinto, 3000-075 Coimbra, Portugal; 2 Medical Genetics Department, Hospital Pediátrico de Coimbra (HPC), ULS Coimbra, Coimbra, Portugal; 3 Faculty of Medicine, University of Coimbra (FMUC), Coimbra, Portugal; 4 Clinical Academic Center of Coimbra (CACC), Coimbra, Portugal; 5 Otolaringology Department, ULS Gaia e Espinho, Gaia, Portugal

**Keywords:** Inherited retinal diseases, Hearing impairment, Ophthalmic genetics, Genotype, Deaf-blindness

## Abstract

**Background:**

Syndromic genetic disorders affecting vision can also cause hearing loss, and Usher syndrome is by far the most common etiology. However, many other conditions can present dual sensory impairment. Accurate diagnosis is essential for providing patients with genetic counseling, prognostic information, and appropriate resources. This study aimed to describe the genetic profile of combined inherited deaf-blindness in Portugal.

**Methods:**

This was a cross-sectional study conducted at a tertiary hospital in Portugal. Patients were identified through the national, web-based inherited retinal dystrophies registry (IRD-PT, retina.com.pt). Demographics, clinical, and genetic data were retrieved from individual patient records. Genetic variants were classified according to the American College of Medical Genetics and Genomics; only likely pathogenic or pathogenic variants were considered relevant for solved cases.

**Results:**

Eighty-four patients (58.3% males; mean age 40.0 ± 17.9 years) from 71 families were included. Usher syndrome was the most frequent etiology (71.4%) followed by Polyneuropathy, hearing loss, ataxia, retinitis pigmentosa, and cataract syndrome (6.0%), Autosomal dominant optic atrophy plus (4.8%) and cone-rod dystrophy and hearing loss (4.8%). Other less frequent etiologies included Alport syndrome (2.4%), Mitochondrial myopathy, encephalopathy, lactic acidosis, and stroke-like episodes (2.4%), Heimler syndrome (2.4%), Senior-Loken syndrome (1.2%), Waardenburg syndrome (1.2%), Maternally inherited diabetes and deafness (1.2%), and Stickler syndrome (1.2%). The overall diagnostic yield of deleterious variants in our deaf–blind cohort was 73.2%. A total of 55 genetic variants were identified across 16 different genes; 11 of these variants are novel and herein reported for the first time.

**Conclusions:**

This is the first study to describe the genetic profile of patients with dual sensory impairment in Portugal, highlighting the genetic heterogeneity associated with inherited deaf-blindness. Usher syndrome was the most prevalent cause in this cohort. Nevertheless, several other less frequent causes must also be considered. This study showed a high diagnostic yield and reported 11 novel genetic variants, thereby contributing to expand the mutational spectrum of these conditions.

## Introduction

Dual sensory impairment of the visual and auditory systems can be caused by a range of conditions. In developed countries, most cases have a genetic basis, with Usher syndrome (USH) accounting for approximately 50% of inherited deaf- blindness [1]. However, other genetic causes of dual sensory impairment may present inherited retinal/optic nerve disease phenotypes and hearing loss (HL), sometimes overlapping with USH. These include other ciliopathies (e.g. Senior-Loker syndrome), *cone-rod dystrophy and hearing loss* or *Polyneuropathy, hearing loss, ataxia, retinitis pigmentosa, and cataract* (PHARC) syndrome [1,2]. The clinical and genetic heterogeneity of inherited retinal dystrophies/degenerations (IRD) makes the molecular diagnosis of combined visual and HL extremely challenging [1,3]. Thus, deep phenotyping complemented by a thorough genetic evaluation is essential to establish a final diagnosis and attempt at genotype–phenotype correlations [4]. Additionally, an accurate and early diagnosis of deaf-blindness syndromes is paramount to provide each patient and their family with the appropriate resources regarding symptom management, genetic counseling, and prognosis [1,4]. Furthermore, genetic profiling contributes to the generation of reference population-based data and opens avenues for inclusion in clinical trials or access to new therapies.

The purpose of this study was to characterize the genetic spectrum of inherited deaf-blindness in Portugal.

## Methods

### Study design

This cross-sectional study was conducted at the largest IRD referral center in Portugal – Hospitais da Universidade de Coimbra, ULS Coimbra, Coimbra, Portugal. Consecutive patients with a clinical diagnosis of combined deaf-blindness and enrolled in the national, web-based IRD registry (IRD-PT, retina.com.pt) [5] until November 2023 were included in the study. The study was approved by the local Ethics Committee and followed the tenets of the Declaration of Helsinki for biomedical research.

### Clinical/demographic features

Clinical and demographic information was collected from each individual patient file, including age, sex, district of residence, symptoms, family history, presence of consanguinity, age of ophthalmic and hearing symptoms onset, presence of ocular and systemic comorbidities, and history of electronic hearing devices (hearing aids or cochlear implants). All patients underwent a complete ophthalmological evaluation in the cross-sectional visit including best corrected visual acuity (BCVA) with Early Treatment Diabetic Retinopathy Study (ETDRS) letters, dilated fundus examination, and multimodal retinal imaging ultra-widefield color fundus photographs (UWF-CFP), UWF fundus autofluorescence (UWF-FAF), spectral domain optical coherence tomography (SD-OCT), and Humphrey visual field testing. An otorhinolaryngology examination was performed in each patient and hearing was assessed with an audiogram in older children/adults and with otoacoustic emissions and/or brainstem auditory evoked potentials in newborns/younger children.

### Genetic testing

Peripheral blood samples were collected, and genomic DNA was isolated using a DNA extraction and purification kit based on the manufacturer’s protocol. A clinically-oriented next-generation sequencing (NGS) approach was used, comprising whole-exome sequencing (WES) or WES-based NGS panels with copy number variation (CNV) screening, complemented by multiplex ligation-dependent probe amplification (MLPA), when necessary. Mitochondrial DNA sequencing was performed to confirm certain diagnoses (e.g. Maternity Inherited Diabetes and Deafness, MIDD). Also, whenever possible, segregation analysis was performed on available family members. Identified genetic variants were classified according to the American College of Medical Genetics and Genomics (ACMG) standards and guidelines for the interpretation of sequence variants [6]. Genetic counseling provided by a medical geneticist was granted to all probands/families. Cases were considered solved in the presence of class IV (likely pathogenic) or V (pathogenic) variants. Among the unsolved cases, those harboring variants of uncertain significance (VUS) in a gene associated with the phenotype were considered *likely solved*, while those showing no clinically relevant variants were considered *truly unsolved*.

### Statistical analysis

Descriptive statistics were used to summarize demographics, clinical, and imaging characteristics. Statistical significance was defined as *p* < 0.05. All statistical analysis was performed using IBM SPSS Statistics 29 for Windows.

## Results

### Clinical/demographic features

**Fig. 1:**
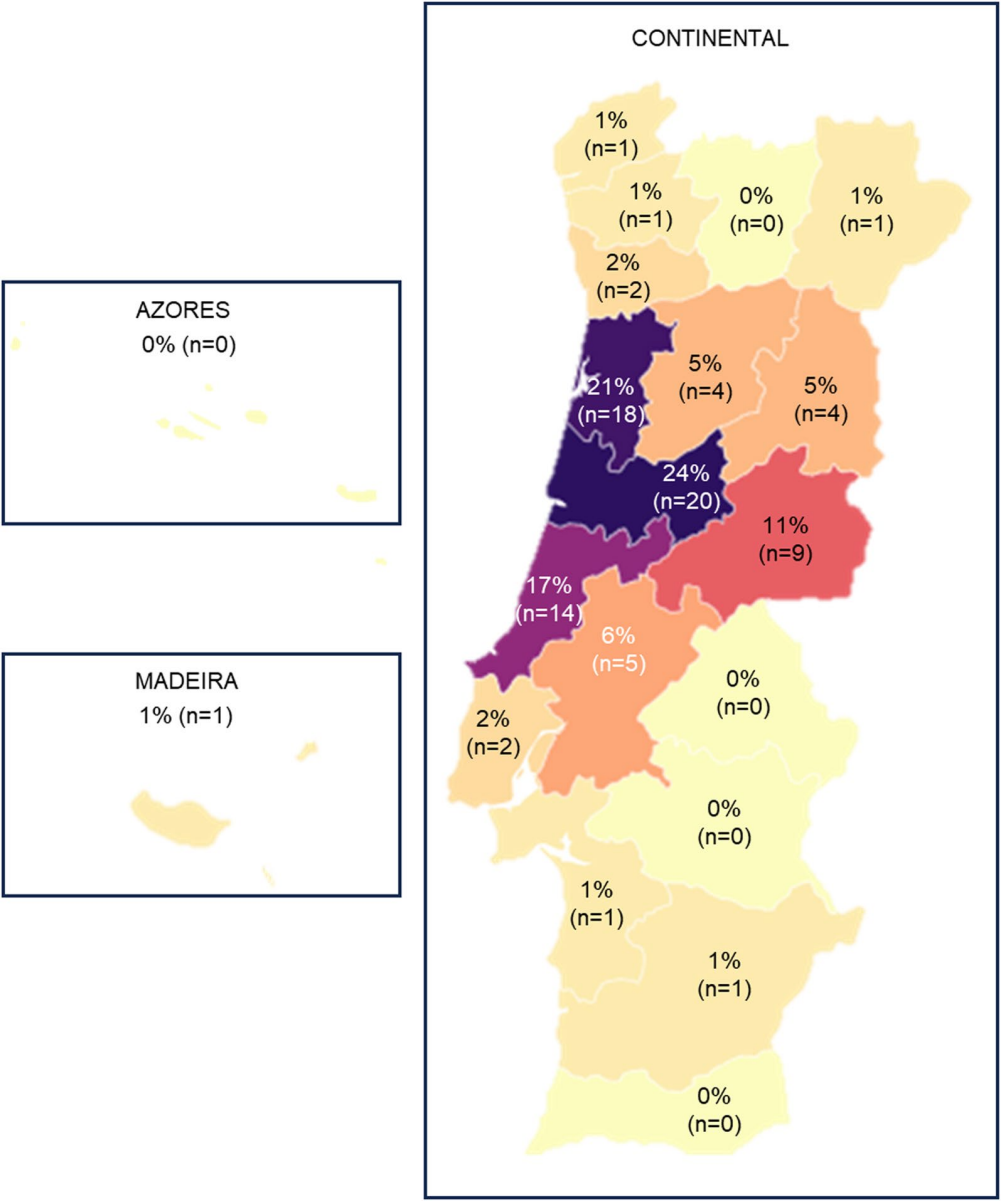
Cohort distribution by district of residence (data presented per patient)

A total of 84 patients (71 families) with a clinical diagnosis of inherited deaf-blindness were included. Most patients were males (58.3%, n = 49), and the mean age at molecular diagnosis was 40.0 ± 17.9 years (range 5–74 years). Family history of the disease was present in 43.7% of our cohort (n = 31 families), while 38.0% (n = 27) of families reported consanguinity. All patients were followed for a median period of 80 months (interquartile range 12–110 months). The demographic characterization of the cohort is presented in Table 1, while the cohort distribution *per* district of residence is presented in Fig. 1.

**Table 1:** Demographic and clinical characterization of the cohort

	Mean	SD
Age at molecular diagnosis (y)	40	17.89
Follow up (m)	79.71	93.87

	N	%
**Male**	49	58.33
**Family History**	44	52.38
**Consanguinity**	34	40.48
*Age of ophthalmic symptom onset*		
≤ 5 years	3	3.57
6–10 years	15	17.86
11–20 years	21	25.00
21–30 years	11	13.10
31–50 years	12	14.29
> 50 years	1	1.19
Unknown	21	25.00
Hearing device	67	79.76
Hearing aids	50	74.63
Cochlear implants	16	19.05
*Age of hearing symptom onset*		
Congenital	22	26.19
Infancy	14	16.67
School-age (5–17 years)	19	22.62
Adult (18–40 years)	17	20.24
Late adult (> 40 years)	7	8.33
Unknown	5	5.95

**Fig. 2:**
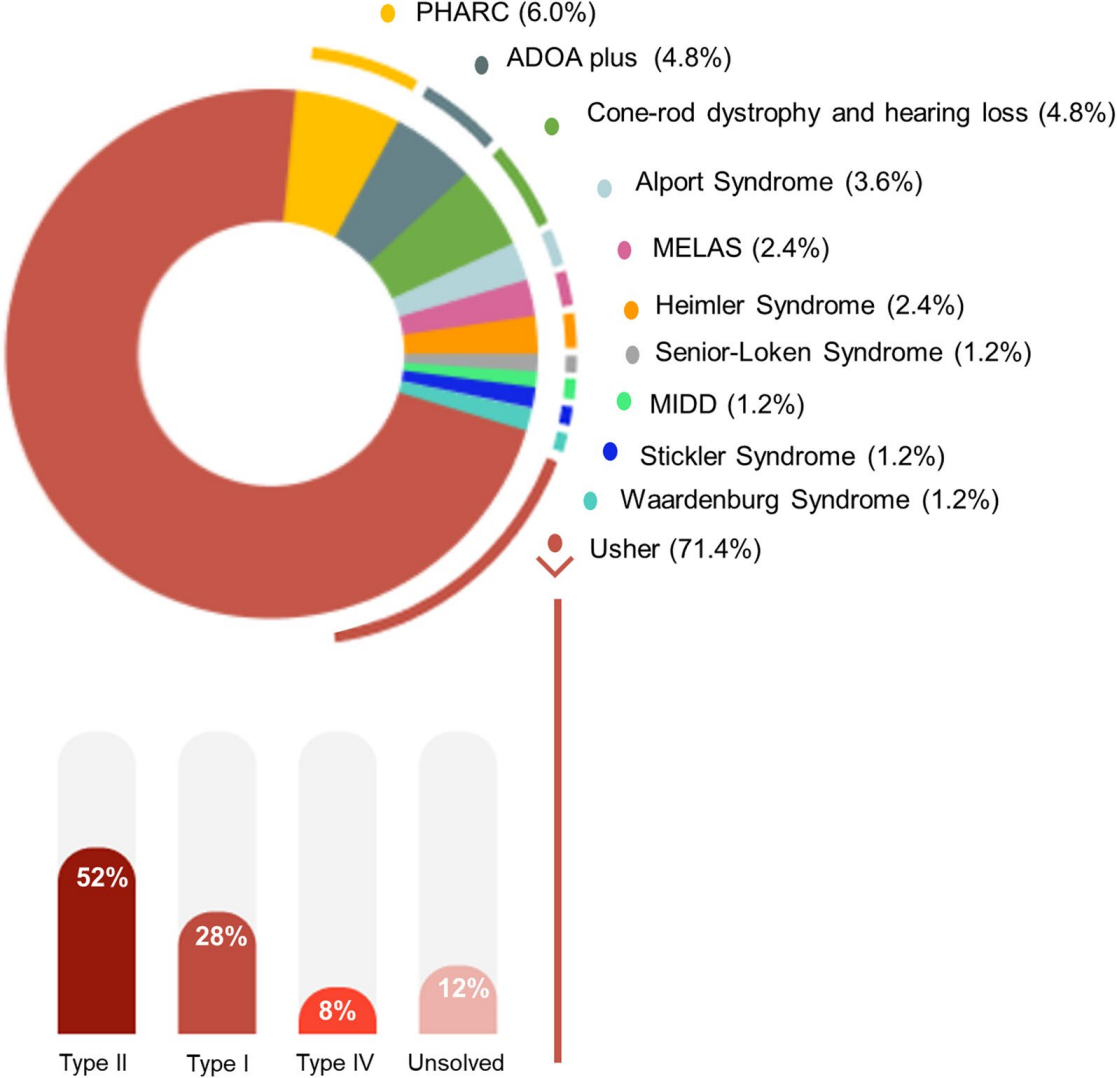
Cohort diagnosis distribution (percentage per patient). ADOA plus—Autosomal dominant optic atrophy plus; MELAS—Mitochondrial myopathy, encephalopathy, lactic acidosis, stroke-like episode; PHARC—Polyneuropathy, hearing loss, ataxia, retinitis pigmentosa, cataract syndrome; MIDD—Maternally inherited diabetes and deafness

USH was the most frequent etiology (71.4%, n = 60 patients, 52 families) followed by PHARC syndrome (6.0%, n = 5, 3 families), Autosomal dominant optic atrophy plus (ADOA plus) (4.8%, n = 4, 2 families) and cone-rod dystrophy and hearing loss (4.8%, n = 4, 4 families). Other less frequent etiologies included Alport syndrome (3.6%, n = 3, 3 families), Mitochondrial myopathy, encephalopathy, lactic acidosis, and stroke-like episodes (MELAS) (2.4%, n = 2, 2 families), Heimler syndrome (2.4%, n = 2, 1 family), Senior-Loken syndrome (1.2%, n = 1, 1 family), Waardenburg syndrome (1.2%, n = 1, 1 family), MIDD (1.2%, n = 1, 1 family), and Stickler syndrome (1.2%, n = 1, 1 family) (Fig. 2). Regarding Usher syndrome, type II was the most frequently encountered (51.7%, n = 31 patients, 25 families), followed by type I (28.3%, n = 17 patients, 16 families) and type IV (8.3%, n = 5 patients, 4 families). Seven patients (7 families) with a clinical diagnosis of USH remained genetically unsolved.

### Ophthalmic findings

The reported visual acuity ranged from 20/20 Snellen equivalent to no light perception. The mean baseline BCVA for this cohort was 58.8 ETDRS letters (Snellen equivalent ~ 20/63), declining to 50.5 ETDRS letters (Snellen equivalent ~ 20/100) at the last follow-up (median 80 months; IQR 12–110 months) (*p* < 0.001).

Age of ophthalmic disease onset, defined as the first instance of ophthalmic-attributable symptoms is presented in Table 1, with most patients reporting the beginning of visual symptoms before 30 years of age. The most frequently observed phenotypes were rod-cone dystrophy (80.95%, n = 68 patients), cone-rod dystrophy (4.76%, n = 4 patients), macular dystrophy (7.14%, n = 6 patients), and optic neuropathy (4.76%, n = 4 patients).

### Hearing findings

The majority of patients (60.7%, n = 51) reported HL before adult age, while thirty-four patients (40.1%) presented with prelingual HL. Most patients (74.6%, n = 50) had an electronic hearing device, with 19.1% (n = 16) of these having cochlear implants.

All patients with Usher type 1 and Heimler syndrome presented with prelingual HL, while Usher type 4, PHARC, and MELAS manifested HL during adulthood. The hearing impairment by age of symptoms and diagnosis are presented in Table 2.

**Table 2:** Age of hearing onset per diagnosis of all cases (percentage per patient)

	Congenital	Infancy	School-age (5–17 y)	Adult (18–40 y)	Late adult (> 40 y)	Unknown
*Usher*						
Type 1	13 (61.9%)	4 (30.8%)				
Type 2	5 (23.8%)	7 (53.8%)	11 (64.7%)	5 (26.3%)	1 (25%)	3 (75%)
Type 4				3 (15.8%)	2 (50%)	
PHARC			1 (5.9%)	4 (21.1%)		
ADOA plus			1 (5.9%)	2 (10.5%)	1 (25%)	
Cone-rod dystrophy and hearing loss		1 (7.7%)	1 (5.9%)	2 (10.5%)		
Alport syndrome		1 (7.7%)	2 (11.8%)			
MELAS				2 (10.5%)		
Heimler syndrome	2 (9.5%)					
Senior-Loken syndrome				1 (5.3%)		
MIDD			1 (5.9%)			
Waardenburg syndrome	1 (4.8%)					
Stickler syndrome						1 (25%)
Total	21	13	17	19	4	4

### Genetic findings

Fifty patients (59.5%) were referred to genetic counseling and molecular diagnosis after consultation with an Ophthalmologist. Otorhinolaryngology accounted for 16.7% (n = 14) of genetic referrals, while other specialties such as Pediatrics, Nephrology, Neurology, and Endocrinology contributed to the remaining referrals.

Disease-causing variants were identified in 52/71 families, resulting in an overall diagnostic yield of 73.2%. Further details on the diagnostic yield and all involved genes per diagnosis are provided in Table 3. A total of 55 unique variants were identified across 16 genes. Eleven variants are novel and herein reported for the first time. These were found in *USH2A, MYO7A, CEP250, ARSG, COL4A5, CDH23, OPA1, LPL/SDCCAG8*, and *COL11A1* genes. A detailed description of all identified genetic variants is presented in Table 4.

**Table 3:** Diagnostic yield and causative gene of combined IRD and HI (data presented per family)

Diagnosis	Genetic testing (N, %)			Gene	N (%)
	Solved	Unsolved	Total		
Usher syndrome	48 (92.3%)	4 (7.7%)	52 (100%)	*USH2A*	22 (42.3)
				*MYO7A*	12 (23.1)
				*ADGRV1*	6 (11.5)
				*ARSG*	4 (7.7)
				*USH1G*	1 (1.9)
				*CDH23*	3 (5.8)
				Unsolved	4 (7.7)
PHARC	3 (100%)	0 (0%)	3 (100%)	*ABHD12*	3 (100)
Cone-rod dystrophy and hearing loss	2 (50%)	2 (50%)	4 (100%)	*CEP250*	2 (50)
				Unsolved	2 (50)
Alport syndrome	3 (100%)	0 (0%)	3 (100%)	*COL4A5*	2 (75)
				*COL4A4*	1 (25)
Heimler syndrome	1 (100%)	0 (0%)	1 (100%)	*PEX1*	2 (100)
Waardenburg syndrome	1 (100%)	0 (0%)	1 (100%)	*MITF*	1 (100)
Senior-Loken	1 (100%)	0 (0%)	1 (100%)	*SDCCAG8*	1 (100)
ADOA plus	2 (100%)	0 (0%)	2 (100%)	*OPA1*	2 (100)
MELAS	2 (100%)	0 (0%)	2 (100%)	*MT-TL1*	2 (100)
MIDD	1 (100%)	0 (0%)	1 (100%)	*MT-TL1*	1 (100)
Stickler syndrome	1 (100%)	0 (0%)	1 (100%)	*COL11A1*	1 (100)
TOTAL	65 (91.6%)	6 (8.5%)	71 (100%)		71 (100)

*ADOA plus* Autosomal dominant optic atrophy plus, *MELAS* Mitochondrial myopathy, encephalopathy, lactic acidosis, stroke-like episode, *PHARC* Polyneuropathy, hearing loss, ataxia, retinitis pigmentosa, cataract syndrome, *MIDD* Maternally inherited diabetes and deafness

**Table 4:** Genetic data of disease-causing variants

Gene	Variant	Protein	Classification	Phenotype	Patients	Families	First Report
*USH2A* (NM_206933.4)	c.10712C > T	p.(Thr3571Met)	PATHOGENIC	Usher 2	1	1	PMID: 17,085,681
	c.7932G > A	p.(Trp2644*)	PATHOGENIC	Usher 2	1	1	PMID: 10,729,113
	c.(7300 + 1_7301-1)_(9371 + 1_9372-1)del		LIKELY PATHOGENIC	Usher 2	4	3	PMID: 38,189,974
	c.11232-2A > G	p.?	PATHOGENIC	Usher 2	1	1	This study
	c.11754G > A	p.(Trp3918*)	PATHOGENIC	Usher 2	1	1	PMID: 10,729,113
	c.907C > A	p.(Arg303Ser)	LIKELY PATHOGENIC	Usher 2	2	2	PMID: 14,970,843
	c.1879C > T	p.(Gln627*)	LIKELY PATHOGENIC	Usher 2	1	1	PMID: 38,189,974
	c.5278del	p. (Asp1760Mefts*10)	PATHOGENIC	Usher 2	2	2	PMID: 10,729,113
	c.11156G > A	p.(Arg3719His)	PATHOGENIC	Usher 2	1	1	PMID: 20,507,924
	c.28091G > A	p.?	PATHOGENIC	Usher 2	3	2	PMID: 10,729,113
	c.6657 + 4A > G	p. ?	VUS	Usher 2	2	1	This study
	c.2276G > T	p.(Cys759Phe)	PATHOGENIC	Usher 2	1	1	PMID: 1,968,399
	c.9304C > T	p.(Gln3102*)	PATHOGENIC	Usher 3	1	1	PMID: 10,729,113
	c.920_923dup	p.(His308Glnfs*16)	PATHOGENIC	Usher 2	4	4	PMID: 18,641,288
	c.(?_−1)_(784 + 1_785-1)	p.?	VUS	Usher 2	1	1	This study
	c.2209C > T	p.Arg737*	PATHOGENIC	Usher 2	1	1	PMID: 17,296,898
	c.2299delG	p.(Glu767Serfs*21)	PATHOGENIC	Usher 2	1	1	PMID: 9,624,053
	c.5329C > T	p.(Arg1777Trp)	LIKELY PATHOGENIC	Usher 2	1	1	PMID: 22,135,276
	c.1214del	p. (Asn405Ilefs*3)	PATHOGENIC	Usher 2	2	2	PMID: 16,098,008
	c.14911C > T	p.(Arg4971)	PATHOGENIC	Usher 2	2	1	PMID: 10,729,113
*USH1G* (NM_173477.5)	c.183 T > A	(p.Cys61*)	LIKELY PATHOGENIC	Usher 1	1	1	PMID: 38,189,974
*MYO7A* (NM_000260.4)	c.5510 T > A	p(Leu1837His)		Usher 1	2	2	PMID: 36,909,829
	c.397dup	p.(His133Profs*7)	PATHOGENIC	Usher 1	1	1	PMID: 21,569,298
	c.6439-1G > A	p. ?	LIKELY PATHOGENIC	Usher 1	1	1	PMID: 16,199,547
	c.5743_574del	p. ?	LIKELY PATHOGENIC	Usher 1	1	1	PMID: 38,189,974
	c.1529 T > C	p. Ile510Thr	LIKELY PATHOGENIC	Usher 1	2	1	PMID: 38,189,974
	c.999 T > G	p.Tyr333Term	PATHOGENIC	Usher 1	1	1	PMID: 8,900,236
	c.1847G > A	p.(Ard616Gln)	VUS	Usher 1	1	1	This study
	c.4489G > C	p.(Gly1497Arg)	LIKELY PATHOGENIC	Usher 1	4	3	PMID: 27,460,420
	c.3508G > A	p.(Glu1170Lys)	LIKELY PATHOGENIC	Usher 1	1	1	PMID: 10,425,080
	c.6026C > A	p.(Ala2009Asp)	LIKELY PATHOGENIC	Usher 1	1	1	PMID: 27,460,420
*ABHD12* (NM_001042472.3)	c.1054C > T	p.(Arg352*)	PATHOGENIC	PHARC	1	1	PMID: 20,797,687
	c.728G > A	p. Trp243*	PATHOGENIC	PHARC	2	1	PMID: 38,189,974
*PEX1* (NM_000466.3)	c.2528G > A	p.(Gly843Asp)	PATHOGENIC	Heimler syndrome	2	1	PMID: 9,398,847
*CEP250* (NM_007186.6)	c.3175_3176del	p.(Ser1060Thrfs*15)	PATHOGENIC	Cone & Rod dystrophy plus hearing loss	1	1	This study
	c.4006C > T	p.(Arg1336*)	LIKELY PATHOGENIC	CORD	1	1	PMID: 30,459,346
*ADGRV1* ( (NM_032119.4)	c.6515C > G	p.(Ser2172*)	LIKELY PATHOGENIC	Usher 2	1	1	PMID: 38,189,974
	c.17668_17669del	p.Met5890Valfs*10	PATHOGENIC	Usher 2	3	3	PMID: 21,569,298
	c.7336del	p.(Glu2446Asnfs*21)	PATHOGENIC	Usher 2	1	1	PMID: 38,189,974
	c. (17,019 + 1_17020-1)_(17,856 + 1_17857-1)dup		LIKELY PATHOGENIC	Usher 2	1	1	PMID: 38,189,974
*ARSG* (NM_001267727.2)	c.1326del	p. (Ser443Alafs*12)	VUS	Usher 4	3	2	PMID: 33,300,174
	c.1150C > T	p.(Arg384Trp)	VUS	Usher 4	1	1	This study
	c.338G > A	p.(Gly113Asp)	LIKELY PATHOGENIC	Usher 4	1	1	PMID: 33,300,174
*COL4A5* (NM_033380.3)	c.(609 + 1_610-1)_(998_?)del		LIKELY PATHOGENIC	Alport	1	1	This study
	c.761_762del	p. (Glu254Valfs*11)	LIKELY PATHOGENIC	Alport	1	1	PMID: 8,648,925
*COL4A4* (NM_000092.5)	c.4449_4450dup	p.(Met1484Thrfs*69)	PATHOGENIC	Alport	1	1	PMID: 29,873,249
*CDH23* (NM_022124.6)	c.3579 + 2 T > C	p. ?	PATHOGENIC	Usher 1	1	1	PMID: 11,138,009
	c.6049 + 1G > A	c.IVS45 + 1G > A		Usher 1	1	1	PMID: 8,894,709
	c.768 + 2 T > A		LIKELY PATHOGENIC	Usher 1	1	1	This study
	c6319C > T	p.(Arg2107*)		Usher 1	1	1	PMID: 11,090,341
*OPA1* *(NM_130837.3)*	c.904A > T	p.(Thr302Ser)	VUS	ADOA	2	1	This study
*MITF* *(NM_001354604.2)*	c.781C > T	p.(Gln261*)		Waardenburg type 2 Syndrome	1	1	PMID: 26,512,583
*SDCCAG8* *(NM_006642.5)*	c.397G > T	p.(Glu133*)	LIKELY PATHOGENIC	Senior-Loken	1	1	This study
*MT-TL1*	m.3243A > G		PATHOGENIC	MELAS MIID	2 1	2 1	PMID: 2,268,345 PMID: 8,603,770
*COL11A1* (NM_001854.4)	c.1846G > C	p.(Gly616Arg)	LIKELY PATHOGENIC	Sticker syndrome	1	1	This study

*ADOA plus* Autosomal dominant optic atrophy plus, *MELAS* Mitochondrial myopathy, encephalopathy, lactic acidosis, stroke-like episode, *PHARC* Polyneuropathy, hearing loss, ataxia, retinitis pigmentosa, cataract syndrome, *MIDD* Maternally inherited diabetes and deafness

Among solved cases of combined IRD and HL, different patterns of inheritance were verified. Most families (n = 50, 70.4%) exhibited autosomal recessive (AR), followed by autosomal dominant (AD) in 5.6% of families (n = 4), X-linked in 1 family (1.4%) and mitochondrial DNA-dependent syndromes in three families (4.2%). Among cases with AR inheritance, a single disease-causing variant in homozygosity was identified in 31 families (62.0%), while 12 (24.0%) presented two different variants in compound heterozygosity. A detailed description of the zygosity of all genetic variants across all the families is presented in Table 5.

**Table 5:** Genetic data of disease-causing variants in solved families

	Patients	Zygosity	Gene	Variant # 1	Variant # 2
Usher syndrome					
Family #2	1	HOM	*USH2A*	c10712C > T p.(Thr3571Met)	c10712C > T p.(Thr3571Met)
Family #3	1	HOM	*MYO7A*	c5510T > A p(Leu1837His)	c5510T > A p(Leu1837His)
Family #5	1	HOM	*USH2A*	c.7932G > A p.(Trp2644*)	c.7932G > A p.(Trp2644*)
Family #6	2	HOM	*USH2A*	c.(7300 + 1_7301-1)_(9371 + 1_9372-1)del	c.(7300 + 1_7301-1)_(9371 + 1_9372-1)del
Family #7	1	HOM	*USH1G*	c.183 T > A p.(Cys61*)	c.183 T > A p.(Cys61*)
Family #9	1	HOM	*MYO7A*	c.397dup p.(His133Profs*7)	c.397dup p.(His133Profs*7)
Family #12	1	C. HET	*USH2A*	c.11232-2A > G p.?	c.11754G > A p.(Trp3918*)
Family #15	1	C. HET	*ADGRV1*	c.6515C > G p.(Ser2172*)	c.(17,019 + 1_17020-1)_(17,856 + 1_17857-1) dup
Family #17	1	C. HET	*USH2A*	c.907C > A p.(Arg303Ser)	c.1879C > T p.(Gln627*)
Family #19	1	HOM	*MYO7A*	c.6439-1G > A p.?	c.6439-1G > A p.?
Family #20	1	C. HET	*USH2A*	c.5278del p. (Asp1760Mefts*10)	c.11156G > A p.(Arg3719His)
Family #21	1	HOM	*USH2A*	c.(7300 + 1_7301-1)_(9371 + 1_9372-1)del	c.(7300 + 1_7301-1)_(9371 + 1_9372-1)del
Family #22	2	HOM	*USH2A*	c.2809 1G > A	c.2809 1G > A
Family #23	1	HOM	*MYO7A*	c.5743_574del p.?	c.5743_574del p.?
Family #25	1	C. HET	*USH2A*	c.2276G > T p.(Cys759Phe)	c.9304C > T p.(Gln3102*)
Family #27	1	HOM	*ARSG*	c.1326del p.(Ser443Alafs*12)	c.1326del p.(Ser443Alafs*12)
Family #28	2	C. HET	*MYO7A*	c.1529 T > C p. Ile510Thr	c.4489G > C p.Gly1497Arg
Family #29	1	HOM	*USH2A*	c.920_923dup p.(His308Glnfs*16)	c.920_923dup p.(His308Glnfs*16)
Family #31	1	HOM	*MYO7A*	c.999 T > G p.Tyr333Term	c.999 T > G p.Tyr333Term
Family #32	1	HOM	*ADGRV1*	c.17668_17669del p.Met5890Valfs*10	c.17668_17669del p.Met5890Valfs*10
Family #34	2	HOM	*USH2A*	c.(7300 + 1_7301-1)_(9371 + 1_9372-1)del	.(7300 + 1_7301-1)_(9371 + 1_9372-1)del
Family #36	1	HOM	*ADGRV1*	c.17668_17669del p.(Met5890Valfs*10)	c.17668_17669del p.(Met5890Valfs*10)
Family #38	1	HOM	*ADGRV1*	c.7336del p.(Glu2446Asnfs*21)	c.7336del p.(Glu2446Asnfs*21)
Family #39	1	C. HET	*USH2A*	c.907C > A p.Arg303Ser	c.2209C > T p.Arg737*
Family #40	1	HOM	*ADGRV1*	c.17668_17669del p.(Met5890Valfs*10)	c.17668_17669del p.(Met5890Valfs*10)
Family #42	1	C. HET	*USH2A*	c.2299delG p.(Glu767Serfs*21)	c.5329C > T p.(Arg1777Trp)
Family #43	1	C. HET	*USH2A*	c.920_923dup p.(His308Glnfs*16)	c.1214del p. (Asn405Ilefs*3)
Family #44	1	HOM	*USH2A*	c.920_923dup p.(His308Glnfs*16)	c.920_923dup p.(His308Glnfs*16)
Family #46	2	HOM	*USH2A*	c.14911C > T p.(Arg4971)	c.14911C > T p.(Arg4971)
Family #48	1	HOM	*CDH23*	c.3579 + 2 T > C p.?	c.3579 + 2 T > C p.?
Family #50	1	HOM	*MYO7A*	c.4489G > C p.(Gly1497Arg)	c.4489G > C p.(Gly1497Arg)
Family #51	1	C. HET	*CDH23*	c.6049 + 1G > A c.IVS45 + 1G > A	c6319C > T p.(Arg2107*)
Family #52	1	HOM	*ARSG*	c.338G > A p.(Gly113Asp)	c.338G > A p.(Gly113Asp)
Family #53	1	HOM	*CDH23*	c.768 + 2 T > A	c.768 + 2 T > A
Family #57	1	HOM	*MYO7A*	c.3508G > A p.(Glu1170Lys)	c.3508G > A p.(Glu1170Lys)
Family #58	1	HOM	*USH2A*	c.920_923dup p. (His308Glnfs*16)	c.920_923dup p. (His308Glnfs*16)
Family #63	1	HOM	*USH2A*	c.5278del p.(Asp1760Metfs*10)	c.5278del p.(Asp1760Metfs*10)
Family #65	1	HOM	*MYO7A*	c.4489G > C p.(Gly1497Arg)	c.4489G > C p.(Gly1497Arg)
Family #67	1	C. HET	*MYO7A*	c.5510 T > A p.(Leu1837His)	c.6026C > A p.(Ala2009Asp)
PHARC					
Family #10	1	HOM	*ABHD12*	c.1054C > T p.(Arg352*)	c.1054C > T p.(Arg352*)
Family #26	2	HOM	*ABHD12*	c.728G > A p. Trp243*	c.728G > A p. Trp243*
Heimler Syndrome					
Family #13	2	HOM	*PEX1*	c.2528G > A p.(Gly843Asp)	c.2528G > A p.(Gly843Asp)
Cone-rod dystrophy and hearing loss					
Family #14	1	HOM	*CEP250*	c.3175_3176del p.(Ser1060Thrfs*15)	c.3175_3176del p.(Ser1060Thrfs*15)
Family #35	1	HOM	*CEP250*	c.4006C > T p.(Arg1336*)	c.4006C > T p.(Arg1336*)
Alport syndrom					
Family #33	1	HEMI	*COL4A5*	c.(609 + 1_610-1)_(998_?)del	N/A
Family #49	1	HEMI	*COL4A5*	c.761_762del p. (Glu254Valfs*11)	N/A
Family #71	1	HEMI	*COL4A4*	c.4449_4450dup p.(Met1484Thrfs*69)	N/A
Waardenburg syndrome					
Family #56	1	HET	*MITF*	c.781C > T p.(Gln261*)	N/A
Senior-Loken syndrome					
Family #68	1	HOM	*SDCCAG8*	c.644G > A p.(Gly215Glu)	c397G > T p.(Glu133*)
Stickler syndrome					
Family #70	1	HET	*COL11A1*	c.1846G > C p.(Gly616Arg)	N/A

*C. HET* Compound Heterozygous, *HOM* Homozygous, *HEMI* Hemizygous, *PHARC* Polyneuropathy, hearing loss, ataxia, retinitis pigmentosa, cataract syndrome, *N/A* Not applicable

Among the unsolved cases (19 families), 12 families were subclassified as *partially solved*: 9 families (12.7%) presented with a VUS, while 3 families (4.3%) harbored one pathogenic/likely pathogenic variant and a VUS in genes associated with the phenotype. The majority were in recessively inherited genes (n = 4 in *USH2A*, n = 2 in *ARSG*, n = 2 in *MYO7A*, n = 1 in *ADGRV1,* and n = 1 in *ABHD12*), and two in *OPA1* gene.

## Discussion

This study represents the first detailed analysis of the genetic basis of inherited deaf-blindness in Portugal. By uncovering 11 novel variants, it provides valuable new insights into the unique genetic underpinnings of dual sensory impairment and highlights the critical role of genetic testing for more accurate diagnoses and personalized care.

Inherited retinal/optic nerve disease displays remarkable allelic and locus diversity. Previous studies have demonstrated that disease-causing variants in genes involved in inherited deaf-blindness can cause variable clinical phenotypes [1–4], thus adding another layer of complexity to the diagnosis.

Although USH is the most common form of inherited dual sensory impairment, there is a variety of hereditary, non-hereditary, and independent causes [1,7].This underscores the importance of an early and accurate diagnosis. Not surprisingly, USH was the most frequent diagnosis in our cohort. However, non-USH genetic causes of deaf-blindness accounted for 28.5% of patients (18 families), a figure somewhat higher than previously reported by Bahena et al [1]. The overall distribution of dual sensory impairment causes in our cohort was heterogeneous but consistent with a recent review of the most common genetic causes of inherited vision and hearing loss [8]. Detailed phenotyping and thorough assessment of the medical and family history were essential to suspect etiologies such as PHARC or Heimler syndrome, highlighting the importance of a multidisciplinary approach [1,2,9].

Recent improvements in genome sequencing techniques have considerably advanced the molecular diagnosis of IRDs. Using state-of-the-art genetic testing, we achieved an overall diagnostic yield of 73.2%, which exceeds 90% when considering the *likely solved* families. This high solving rate is consistent with NGS-based genetic testing of patients with inherited deaf-blindness [1,9,10], suggesting that most genes underlying dual sensory loss have already been identified [1,11,12]. Our cohort identified 55 distinct genetic variants across 16 different genes. Eleven novel disease-causing variants across 9 genes are herein reported for the first time. These new variants were encountered in USH, Alport Syndrome, Cone-rod dystrophy and hearing loss, ADOA plus, Stickler syndrome, and Senior-Loken syndrome. The remaining variants had been previously reported by our group [10] or other European cohorts [13,14].

The most frequently involved genes in USH syndrome in this study were *USH2A* and *MYO7A*, together comprising 65% of solved cases, while *USH1G* was the least frequent (1.8%). This is consistent with a recent meta-analysis of NGS data in the United States [15]. Interestingly, our cohort presented a higher rate of homozygosity (54%) compared to a multicenter European and a French study, which reported 26.0% (n = 111/427) and 11.3% (n = 26/231), respectively [13,14]. This high rate of homozygosity is likely explained by a relatively low population density in Portugal, along with a high percentage of familial consanguinity (38%), as recently reported by our group [10].

Approximately 16% of families in our study had at least one variant of unknown pathogenicity in disease genes that fit the USH phenotype. For the two patients with one pathogenic/likely pathogenic variant and one VUS identified, family studies were not available to help establish a final diagnosis. Bearing in mind that reclassification of VUS may occur over time, all cases are offered clinical follow-up in an yearly basis and are genetically revisited every 2–3 years. This approach can increase the molecular diagnosis rate of IRDs, with a substantial impact on the lives of these patients and their families [16,17]. The same applies to fully unsolved cases, which may eventually be elucidated with the expanding role of targeted adaptive long-read sequencing, as non-coding variants may account for the missing heritability [16].

Establishing the genotype of an IRD is considered an essential component of the diagnostic workup. In this study, a high proportion of patients (n = 50, 59.5%) with presumed IRD were referred for genotyping, following an appointment with an Ophthalmologist. Of those, one-quarter presented with prelingual HI but only had their molecular diagnosis after 40 years of age. A few conclusions can be drawn from these findings. Firstly, most causes of inherited deaf-blindness have age-dependent symptoms, which compels how crucial it is to raise awareness among healthcare professionals of these neuro-sensory disorders to ensure timely referrals to multidisciplinary care [1,3,17]. Molecular diagnosis of patients who are pre-symptomatic for an expected symptom will allow specialized counseling and targeted treatments before symptoms manifest or progress [11,19].This applies especially to children: not only to have access to visual rehabilitation during the appropriate stages of visual development to prevent amblyopia [16,18,19]; but also to those with particularly severe to profound sensorineural hearing loss (SNHL). In the latter, cochlear implants should be offered between 6 and 12 months of age, as implantation during the first year of life is correlated with better language outcomes [20,21]. Secondly, the broader availability of genetic tests warrants the need for well-established referral pathways for patients with inherited deaf-blindness to be managed at expert centers [1,12,17,18,22]. This will allow accurate genetic counseling for patients/families, identification of suitable clinical studies or treatment opportunities, and ultimately improve patient care [12,17–19]. Lastly, genetic profiles of IRDs vary among regions and ethnic groups, underscoring the importance of obtaining reference population-based data [12,17–19].

This study is not exempt of limitations. First, even though we were able to enroll a significant number of patients with rare conditions associated with dual sensory impairment, some Portuguese regions are underrepresented in our cohort. Patients from these regions may be receiving care elsewhere or live in rural areas, which suffer from health access disparities. Second, follow-up time varied widely among patients, precluding a true natural history evaluation. As our national IRD registry^5^ grows, a wealth of information will be gathered, allowing better disease characterization over time.

## Conclusion

This is the first study to comprehensively describe the genetic landscape of inherited deaf-blindness syndromes in Portugal. It establishes population-based reference data and further expands the mutational spectrum of dual sensory impairment by reporting 11 novel variants across 9 different genes associated with inherited deaf-blindness. These findings emphasize the importance of establishing a final molecular diagnosis as inheritance patterns, phenotypes, management, and prognosis significantly differ between the many possible causes of dual sensory impairment.

## Data Availability

The data that support the findings of this study are not openly available due to reasons of sensitivity and are available from the corresponding author, upon reasonable request. Data are located in controlled access data storage at CHUC.
